# Review on Quasi One-Dimensional CdSe Nanomaterials: Synthesis and Application in Photodetectors

**DOI:** 10.3390/nano9101359

**Published:** 2019-09-23

**Authors:** Weifeng Jin, Luodan Hu

**Affiliations:** Key Laboratory of Optoelectronic Technology & Systems of Ministry of Education, College of Optoelectronic Engineering, Chongqing University, Chongqing 400044, China; 20152504@cqu.edu.cn

**Keywords:** CdSe, quasi one-dimensional nanomaterials, synthesis, photodetector

## Abstract

During the past 15 years, quasi one-dimensional (1D) Cadmium Selenide (CdSe) nanomaterials have been widely investigated for high-performance electronic and optoelectronic devices, due to the unique geometrical and physical properties. In this review, recent advancements on diverse synthesis methods of 1D CdSe nanomaterials and the application in photodetectors have been illustrated in detail. First, several bottom-up synthesis methods of 1D CdSe nanomaterials have been introduced, including the vapor-liquid-solid method, the solution-liquid-solid method, and electrochemical deposition, etc. Second, the discussion on photodetectors based on 1D CdSe nanomaterials has been divided into three parts, including photodiodes, photoconductors, and phototransistors. Besides, some new mechanisms (such as enhancement effect of localized surface plasmon, optical quenching effect of photoconductivity, and piezo-phototronic effect), which can be utilized to enhance the performance of photodetectors, have also been elaborated. Finally, some major challenges and opportunities towards the practical integration and application of 1D CdSe nanomaterials in photodetectors have been discussed, which need to be further investigated in the future.

## 1. Introduction

Cadmium selenide (CdSe), one of the group-II–VI compound semiconductors, possesses extraordinary electronic and optoelectronic properties. CdSe can commonly exist in three crystalline structures, namely, wurtzite, zinc blende, and rock-salt. CdSe with a zinc blende structure is unstable and can convert to a wurtzite structure under proper heating conditions, while a rock-salt structure can be observed only under high pressure [1]. Specifically, CdSe usually exhibits unipolar *n*-type conductivity and it is difficult to form *p*-type, because of the self-compensation effect [2,3]. Low-dimensional CdSe nanomaterials possess unique geometrical and physical characteristics and have been widely investigated in both basic science and practical application. Quasi one-dimensional (1D) CdSe nanomaterials mainly include CdSe nanowires (NWs), nanobelts (NBs), and nanotubes (NTs), etc. [4,5,6]. A direct bandgap (~1.74 eV at 300 K for bulk) and a high absorption coefficient near the band edge, make 1D CdSe nanomaterials a good choice for the design in next-generation high-performance photodetectors, solar cells, light-emitting diodes, field-effect transistors, and lasers, etc. [7,8,9,10,11,12].

Photodetectors are one important type of optoelectronic devices, which can transform light signals into electrical signals and have been extensively used in civil and military fields, such as space communication, remote sensing, missile guidance, night vision, motion detection, and so forth. In photodetectors, the advantages of 1D semiconductor nanomaterials compared to their bulk counterparts are as follows: (1) large surface-to-volume ratio can enhance light absorption and guarantee the possibility of surface decoration, which can be used to improve photoresponse [13], (2) anisotropic geometries can be utilized in polarization-sensitive photodetection [14], (3) when the diameters of 1D nanomaterials are in the range of exciton Bohr radius, the size-dependent bandgap can broaden the spectral range of photodetection [15], (4) 1D nanomaterials have superior mechanical characteristics and can be employed in flexible fields [16,17], (5) lower power consumption, and (6) low-dimensional nanomaterials are promising for application in laser-induced change of optical absorption [18].

Up to now, there are few comprehensive reviews focusing on the synthesis of 1D CdSe nanomaterials and their application in photodetectors [19,20]. First, we have introduced and compared several bottom-up methods developed to synthesize 1D CdSe nanomaterials. Then, the recent progresses on photodetectors based on 1D CdSe nanomaterials have been discussed in detail. Finally, some major challenges and opportunities in this field have been analyzed. This work can provide meaningful guidance for the fabrication and design of novel nanoscale photodetectors based on 1D CdSe nanomaterials in the future.

## 2. Synthesis of One-Dimensional (1D) Cadmium Selenide (CdSe) Nanomaterials

Morphologies, crystalline structures, and doping of 1D CdSe nanomaterials have significant influence on the electrical and optoelectrical properties, and great attention has been paid to the synthesis methods. In general, semiconductor nanomaterials can be fabricated with top-down or bottom-up methods [21,22]. Top-down methods usually fabricate bulk materials into desired nanostructures with lithography (ultraviolet, e-beam, or nanoimprint lithography, etc.) and etching processes (reactive ion, wet chemical, or focused ion beam etching, etc.), while bottom-up methods utilize atoms, molecules, or nanostructure units as building blocks to construct nanoscale devices and systems. However, both physical limits and economic factors of current lithography technologies will limit the fabrication and scalability of semiconductor nanomaterials below 10 nm. What is worse, the issues of interfacial lattice mismatch must be taken into account when semiconductor nanomaterials fabricated with top-down methods integrate with diverse substrates. Currently, 1D CdSe nanomaterials are commonly synthesized with bottom-up methods, such as vapor-liquid-solid method, solution-liquid-solid method, or electrochemical deposition, etc. 1D CdSe nanomaterials synthesized with bottom-up methods can integrate with various substrates and maintain their native electrical and optical properties. Therefore, bottom-up methods exhibit great potential and flexibility in synthesizing low-dimensional nanomaterials.

### 2.1. Vapor-Liquid-Solid Method

In 1964, Wagner and Ellis found an important method to fabricate 1D nanostructures, which was called vapor-liquid-solid (VLS) mechanism since it contains gas precursor vapor, liquid alloy droplets, and solid crystallization products [23]. The VLS mechanism consists of three main steps including evaporation of the precursor, initial alloying of the semiconductor-metal catalyst, crystal nucleation, and axial growth. Typically, metal particles are employed as catalysts for the growth of nanomaterials. The diameter of as-synthesized nanomaterials is directly proportional to the size of metal catalyst. Chemical vapor deposition (CVD) is usually used to produce gas precursors, transport them to the surface of molten catalyst droplets in a VLS process, and has gradually become a common technology to synthesize various 1D nanomaterials.

**Figure 1:**
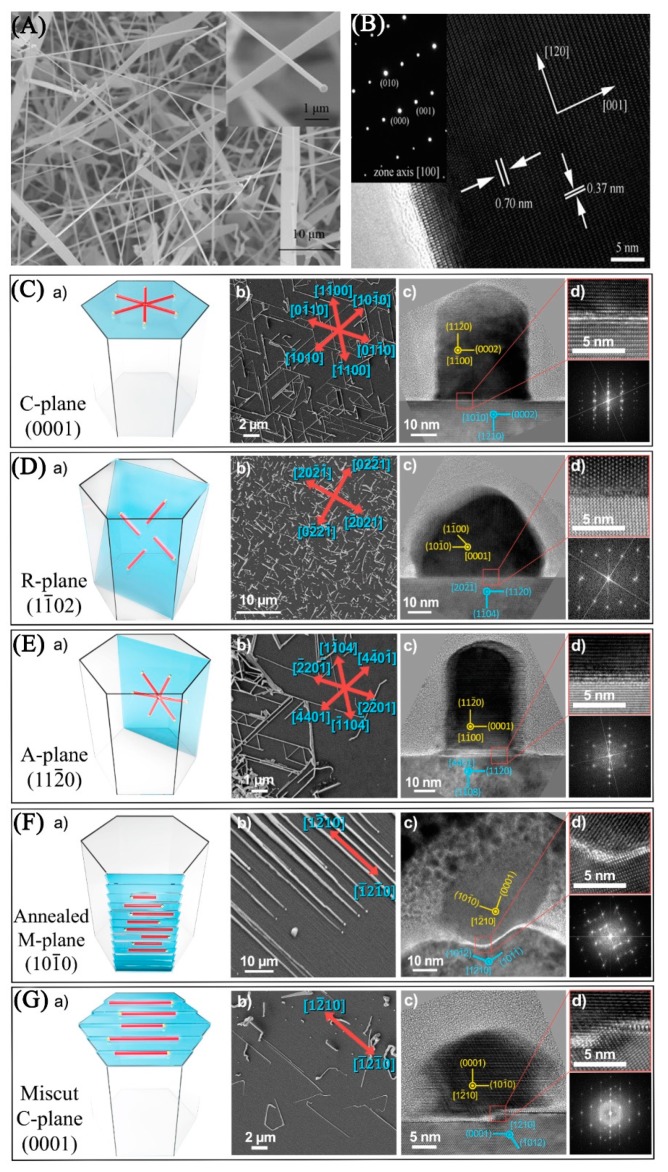
(**A**) Scanning electron microscope (SEM) image and (**B**) high-resolution transmission electron microscope (HRTEM) image of as-synthesized CdSe nanowires (NWs). Reproduced from [11], with permission from the Royal Society of Chemistry, 2014. (**C–G**) Guided growth of horizontal CdSe NWs on different planes of sapphire, for each plane of sapphire: **a**) schematic illustration, **b**) SEM image, **c**) and **d**) HRTEM cross-section image of the guided CdSe NWs. Reproduced from [25], with permission from American Chemical Society, 2017.

Our group synthesized CdSe NWs based on the VLS mechanism via the CVD method with Au as the catalyst [5,6,11]. The morphology and length of NWs could be modulated by controlling the growth temperature, time, and gas pressure, etc. Figure 1A,B shows the scanning electron microscope (SEM) and high-resolution transmission electron microscope (HRTEM) of as-synthesized CdSe NWs, respectively [11]. A catalyst particle could be seen at the end of the NW in the inset of Figure 1A, which was a demonstration of a VLS growth mechanism. The doping concentration of CdSe NWs could be rationally controlled by the introduction of dopant. Lee’s group fabricated CdSe nanostructures with various shapes through a bismuth-assisted VLS process [24]. In their method, bismuth (Bi) and CdSe mixed powders were used as precursors and transferred to Au catalysts by argon via CVD method. CdSe NWs and NBs could be controllably synthesized when the reaction temperature was 650 °C and 850 °C, respectively. Recently, Joselevich et al. reported guided VLS growth of well-aligned horizontal CdSe NWs arrays on five different planes of sapphire substrates (as shown in Figure 1C–G) in which the growth and assembly of NWs were combined into one single step [25]. The growth location, direction, and crystallographic orientation of CdSe NWs were controlled by epitaxial and graphoepitaxial relations with the underlying substrates. Generally, 1D CdSe nanomaterials synthesized with the VLS mechanism have high crystal quality (usually single crystalline) due to high reaction temperature. However, the diameter distribution is wide since controlling the size of catalyst droplets is difficult.

### 2.2. Solution-Liquid-Solid Method

The solution-liquid-solid (SLS) mechanism is somewhat analogous to the VLS mechanism. In a SLS process, the precursor is delivered in solution phase, while it is in vapor phase in the VLS process. In SLS, the metal particles will catalyze the decomposition process of metallo-organic precursor solution at the solution-liquid interface and dissolve the semiconductor components, then promote the supersaturation and crystallization of the semiconductor NWs from the catalyst droplet [26]. The reaction temperature is lower (200–400 °C) in comparison to VLS due to the application of metal catalysts with low melting points, such as Ga (29.8 °C), In (157 °C), Sn (232 °C), and Bi (271 °C) [27,28,29,30]. The low reaction temperature of SLS makes it more advantageous in the synthesis of small diameter nanomaterials with strong quantum confinement effect.

**Figure 2:**
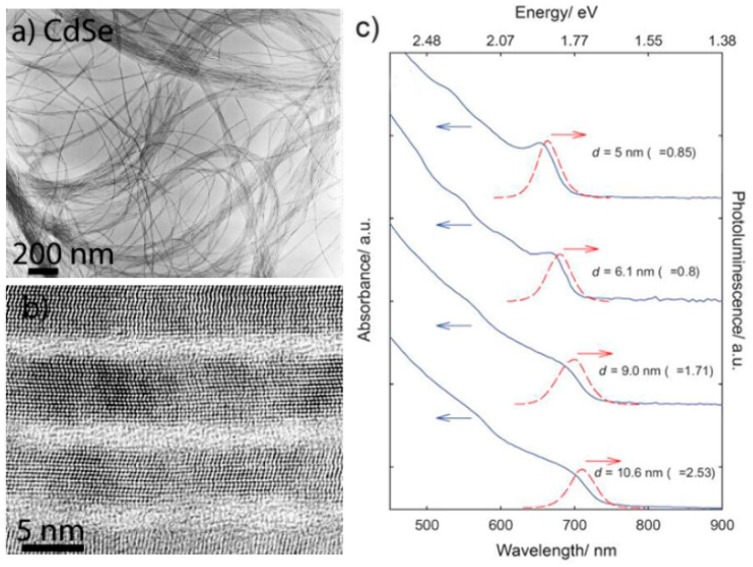
(**a**) Low and (**b**) high resolution TEM image, (**c**) size-dependent absorption and photoluminescence (PL) spectra of CdSe NWs synthesized with the solution-liquid-solid (SLS) mechanism. Reproduced from [34], with permission from Wiley, 2009.

To date, most CdSe NWs synthesized with the SLS mechanism are with Bi-based nanoparticles as catalysts [31,32,33,34]. Buhro et al. fabricated CdSe NWs using Bi nanoparticles as catalysts based on the SLS mechanism [15]. The CdSe NWs were synthesized from cadmium stearate and *n*-R_3_PSe (R is butyl or octyl) in trioctyl phosphine oxide at 240–300 °C. The diameters of NWs were in the range of 5–20 nm and could be controlled by varying the reaction temperature and the size of the catalyst nanoparticle. Another investigation from Li group showed how the diameters of the CdSe NWs depended on the growth of Bi nanoparticles during the SLS process [31]. Since there was a competition between the coagulation of Bi particles and the growth of CdSe NWs, accelerating the growth of the Bi catalysts or slowing the growth of CdSe NWs can lead to larger diameter and vice versa. In order to seek more convenient synthetic methods, Kuno et al. proposed a simplified SLS synthesis method of CdSe NWs, using only Bismuth salt such as BiCl_3_ as a catalyst, instead of pre-synthesized Bi nanoparticles [34]. Figure 2a,b exhibits the transmission electron microscope (TEM) images of as-synthesized CdSe NWs with different diameters and shows apparent quantum confinement effects in the absorption spectrum, as seen in Figure 2c. The diameter could be tuned by simply changing the amount of Bi salt in the reaction. The size distribution of synthesized NWs varied from 13% to 24% and tended to increase with increasing diameter.

### 2.3. Electrochemical Deposition

The electrochemical deposition has remarkable strengths in synthesizing nanomaterials, such as fast growth rate, high uniformity, large cover surface, and inexpensive apparatus [35]. A three-electrode apparatus is usually used in this method, including a reference electrode, a counter electrode, and a working electrode. Appropriate electric potential is applied to the electrolyte-containing precursors, and finally the required nanomaterials are deposited on the working electrode.

**Figure 3:**
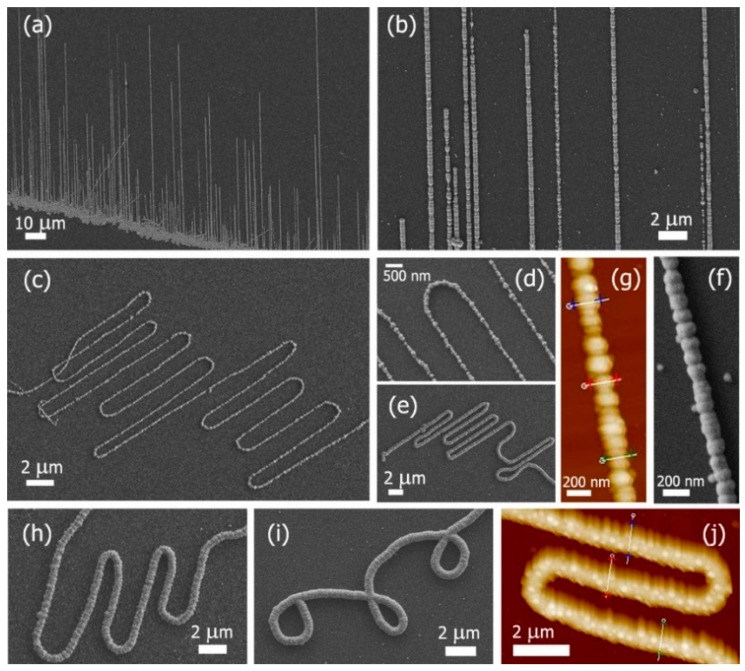
SEM images of CdSe NWs with diverse geometries: (**a**), (**b**), (**f**) straight arrays, (**c**), (**d**), (**e**), and (**h**) thin serpentine, and (**i**) coiled. Atomic force microscope (AFM) images of: (**g**) straight NW, in which heights of marked sections are 87 (blue makers), 84 (red), and 68 nm (green), (**j**) serpentine NW, in which heights of marked sections are 450 (blue), 540 (red), and 500 nm (green). Reproduced from [37], with permission from American Chemical Society, 2012.

Zhang’s group synthesized CdSe NW arrays with various growth orientations by employing the electrochemical deposition using porous alumina as templates (PATs) [36]. They demonstrated that the growth orientation of CdSe NWs could be effectively tuned by simply varying either the nanopore diameter of the PATs or the deposition current density. Joselevich et al. reported a modified electrodeposition method to synthesize polycrystalline CdSe NWs with controlled geometries, such as straight arrays, serpentines, or loops, etc., as seen in Figure 3 [37]. Single-walled carbon nanotubes (SWNTs), which were synthesized via the CVD method, were used as the underlying electrode and template for the selective electrodeposition of CdSe NWs. The pattern of SWNTs could be defined by the patterned iron catalyst, which was fabricated with photolithography and electron-beam evaporation. In the work, the shape of the electrodeposited CdSe NWs could be controlled by the underlying SWNTs. 

### 2.4. Other Methods

**Figure 4:**
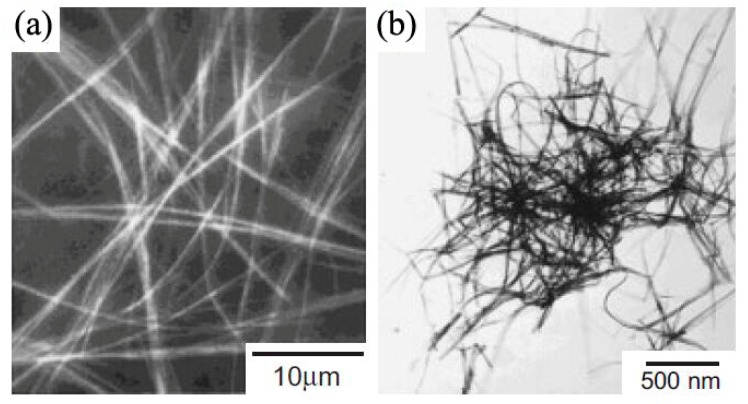
(**a**) SEM image of CdS NWs and (**b**) TEM image of CdSe NWs synthesized with the solvothermal method. Reproduced from [42], with permission from Wiley, 2003.

Solvothermal/Hydrothermal technology refers to a low-temperature and high-pressure chemical reaction process in a closed vessel (such as autoclave) with aqueous or non-aqueous solution as a solvent, and has the advantages of high yield, simple reaction apparatus, and low reaction temperature [38]. Solvothermal/Hydrothermal technology is very suitable for synthesizing 1D CdSe nanomaterials since their morphologies and crystalline phases can be controlled by adjusting monomer concentration, reaction temperature, composition, proportion of precursors, or surfactants, etc. [39,40,41,42]. Tang et al. studied the solvothermal growth process of CdSe and cadmium telluride (CdTe) NWs, which were synthesized through the redox reaction of selenite and tellurite with cadmium salt in the presence of poly (vinyl alcohol) (PVA) in 160–180 °C [40]. In the work, PVA could promote the oriented attachment in the solvothermal process, which resulted in preferred growth of CdSe NWs. Most of as-synthesized CdSe NWs were in the zinc blend structure and could transform from zinc blend to wurtzite structure when the reaction temperature was higher than 170 °C. Qian et al. synthesized CdSe NWs, CdS NWs, and CdS-CdSe core-shell NWs with the solvothermal method [42]. Figure 4 shows the as-synthesized CdS and CdSe NWs. The length of NWs could be as long as 100 μm. It was demonstrated that solvents have significant influence on the morphology and optical property of CdSe NWs.

**Figure 5:**
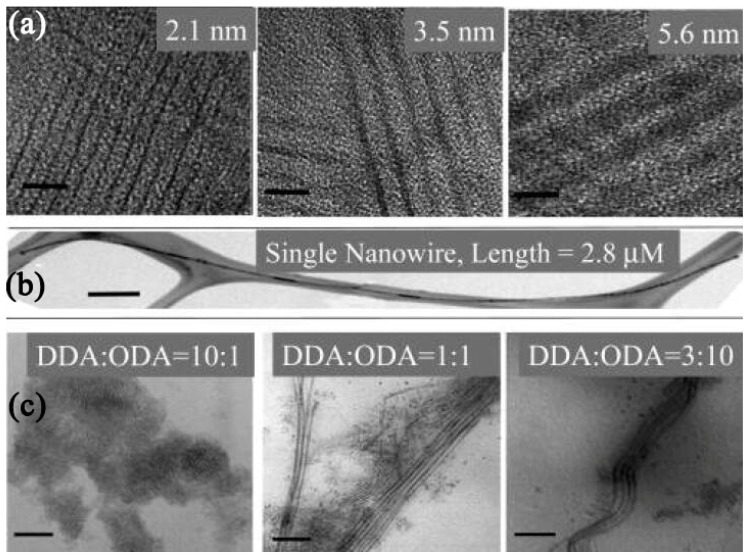
TEM images of (**a**) CdSe NWs with diverse diameters, (**b**) a single CdSe NW, and (**c**) CdSe NWs synthesized with different components of amine. The scale bars of (**a**), (**b**), and (**c**) are 10, 200, and 50 nm, respectively. Reproduced from [44], with permission from American Chemical Society, 2006.

Oriented attachment refers to the approach of attaching existing dot-shaped nanoparticles along a certain crystal orientation to generate NWs [43,44,45]. The collective behavior of nanoparticles and interactions between them are the main determinants of orientation growth. First, nanoparticles find appropriate lattice to match after a series of rotations. Then, the interaction along a specific direction will drive nanoparticles to fuse spontaneously and form linear aggregates. Finally, NWs can be formed via recrystallizing. This method enables excellent uniformity in NW diameter and high aspect ratio. Pradhan’s group utilized this method to synthesize single-crystalline CdSe NWs with tunable diameters in the strong quantum confinement size regime (1.5–6 nm) at relatively low temperatures (100–180 °C) [44]. Figure 5 exhibited the TEM images of as-synthesized CdSe NWs. The length of NWs was a few micrometers. The diameters could be tuned by varying the reaction temperature or using alkylamines (a single type or mixture of two different types of amines) with different chain lengths. Three different growth stages of CdSe NWs were identified, including generation of magic-sized clusters, formation of prewire aggregates, and appearance of solid NWs.

## 3. Application of 1D CdSe Nanomaterials in Photodetectors

### 3.1. Performance Parameters of Photodetectors

Before the introduction of photodetectors, we have summarized some important performance parameters of photodetectors.

(1) Photocurrent (*I*_ph_): defined as the current contribution due to the absorption of signal light.
(2) Dark current (*I*_d_): defined as the output current of photodetectors in the absence of illumination.
(3) Responsivity (*R*): defined as the photocurrent generated per unit power of incident light on the effective area of a photodetector. $R=I_{ph}/P_{in}$, in which *I*_ph_ is the photocurrent, and *P*_in_ is the power of incident light. The unit of *R* is A/W.
(4) External quantum efficiency (*η*): defined as the ratio of the number of photogenerated electron-hole pairs per second to the number of incident photons per second. $\eta =\frac{I_{ph}/q}{P_{in}/h\nu }=\frac{h\nu }{q}R$, in which *h* is the Planck constant, *ʋ* is the frequency of signal light, and *q* is the elementary charge.
(5) Gain (*G*): defined as the number of charges collected by the electrodes for each absorbed incident photon. $G=\frac{\tau }{\tau_{tr}}=\frac{\tau }{l^{2}/\mu V}$, in which *τ* is the lifetime of excess carrier, *l* is the channel length, *τ*_tr_ is the carrier transit time across the channel, *V* is the applied voltage bias, and *μ* is the carrier mobility.
(6) Response time: defined as the time for the photocurrent of a photodetector to increase (decay) from 10% (90%) to 90% (10%) after receiving (removing) signal light, which is also named as rising/falling time.
(7) Specific detectivity (D*): used to evaluate the capability of a photodetector in weak light detection. $D^{*}=\sqrt{AB}/NEP$, in which *A* is the effective area of a photodetector, *B* is the electrical bandwidth, and *NEP* is the signal power that produces a signal-to-noise ratio to be equal to 1. The unit of D* is cm·Hz·^0.5^·W^–1^ (Jones).

### 3.2. Photodetectors Based on 1D CdSe Nanomaterials

Generally, the basic physical mechanism of semiconductor photodetectors can be described as follows: first, electron-hole pairs are generated in semiconductors after the absorption of the above-bandgap photons. Then, photogenerated electron-hole pairs are separated and collected to the electrodes by a built-in or external electric field. Finally, photocurrent is formed in the external circuit. The role of such a separating and collecting electric field can be played by either a built-in electric field (as in photodiodes), or an external voltage bias (as in photoconductors or phototransistors).

#### 3.2.1. Photodiodes

Photodiodes refer to photodetectors based on Schottky, *pn*, or *pin* junctions. Based on band alignments, junctions can be typically divided into four types, including Schottky, type-I, type-II, and type-III junctions. Schottky junctions are composed of metal and semiconductor with certain work function differences [46,47]. Type-I junctions [48,49], in which the conduction band minimum and valence band maximum of the semiconductor with narrower bandgap are placed in between those of the other, have been widely used in fabricating high-efficiency light emitting devices. The typical characteristic of type-III band alignment is the broken gap. Type-III junctions are commonly used to build tunneling diodes or field-effect transistors [50,51,52]. In type-II junctions, both the conduction band minimum and valence band maximum in one semiconductor are higher than those of the other semiconductor [53,54]. In both Schottky junctions and type-II junctions, the built-in electric fields can promote the separation and decrease the recombination rate of photogenerated electrons and holes, which is advantageous for photodetection and photovoltaic devices. Therefore, most photodiodes are based on Schottky junctions or type-II junctions. Photodiodes have some advantages over photoconductors, such as high response speed, low dark current, and possible zero-bias operation due to the built-in electric field.

**Figure 6:**
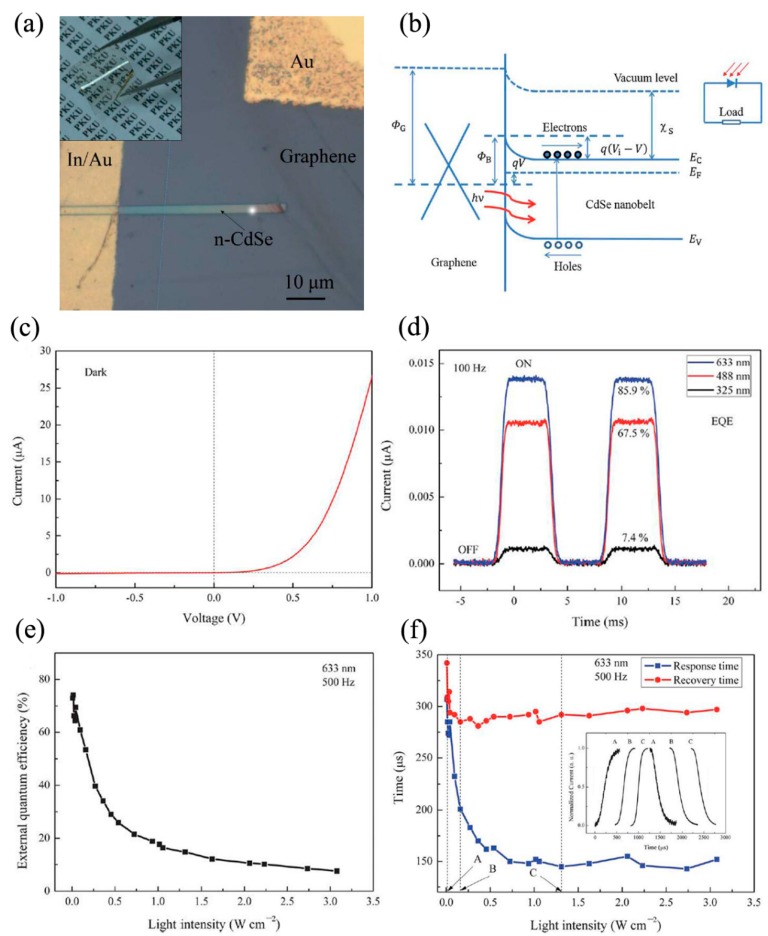
Photodetectors based on CdSe nanobelts (NB)/graphene Schottky junction on a polyethylene terephthalate (PET) substrate: (**a**) the optical image, (**b**) the energy band diagram, (**c**) *I-V* characteristic in dark condition, (**d**) the photoresponse of the device together with the calculated external quantum efficiency (EQE) under different light wavelength, (**e**) the light intensity dependence of EQE, (**f**) the light intensity dependence of the response and recovery times. Reproduced from [16], with permission from the Royal Society of Chemistry, 2013.

Our group fabricated and investigated self-powered photodetectors based on CdSe NB/graphene Schottky junctions on both rigid and flexible substrates for the first time, as seen in Figure 6a,b [16,55]. Compared to traditional electrode materials (such as metal and indium titanium oxide), graphene has many advantages, such as high carrier mobility, flexibility, and light transparency. Typical Schottky junctions exhibited good rectifying behavior with on/off ratio more than 10^3^ under dark conditions, as shown in Figure 6c. The influences of light wavelength and intensity on the external quantum efficiency (EQE) and response speed were discussed. In Figure 6d, EQE was lower for signal light with a shorter wavelength because of shorter light penetration depth in CdSe NB. EQE decreased monotonically as light intensity increased, as shown in Figure 6e. The response and recovery times of such photodetector were about 70 and 137 μs respectively, under 3500 Hz light switching frequency. As seen in Figure 6f, both the response and recovery time decreased first, and then came to constant values as light intensity increased, which might arise from the filling and saturation processes of the hole-traps in CdSe NBs. The performances of flexible photodetectors under various bending conditions have also been investigated. Joselevich et al. reported photodetectors based on self-aligned CdS-CdSe core-shell nanowalls, which were synthesized with a combination of surface-guided VLS horizontal growth and selective-area vapor-solid epitaxial growth [56]. It is worth mentioning that the integration of CdS-CdSe core-shell nanowalls into photodetectors at wafer-scale is without postgrowth transfer, assembly, or etching steps. The response time, responsivity, and gain of photodetectors were about 200 ns, 1.2 × 10^3^ A/W, and 3.8 × 10^3^, respectively. The simultaneous realization of sub-microsecond response and high gain was attributed to high crystalline quality of CdS-CdSe core-shell nanowalls and the quasi-type-II band alignment, which could enhance the separation efficiency of photogenerated carriers.

#### 3.2.2. Photoconductors

Photoconductors, also named as photoresistors, are mainly composed of two Ohmic contact electrodes and certain semiconductor materials between them as a photoactive layer. Photoconductive effect is the main physical mechanism of photoconductors, in which carriers are generated under illumination either by above-bandgap transitions or by transitions involving forbidden-gap energy levels, resulting in an increase in electrical conductivity of semiconductors [57]. Compared to photodiodes, photoconductors are a kind of low-speed and noisy devices, but their simplicity, high responsivity, and gain are attractive features.

**Figure 7:**
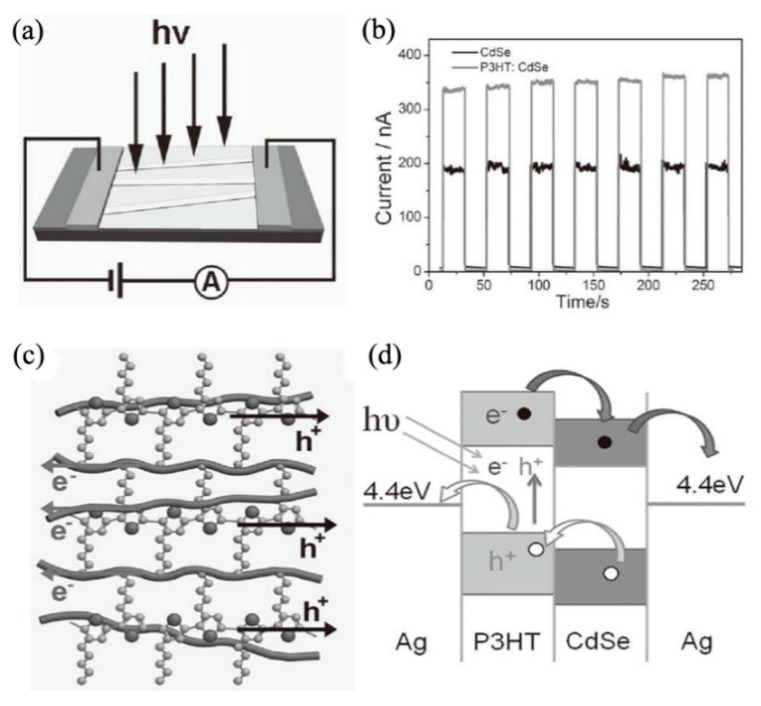
(**a**) Schematic illustration, and (**b**) switching photoresponse of typical CdSe NWs/poly(3-hexylthiophene) (P3HT) hybrid photodetector on a silicon substrate, (**c**) schematic of the CdSe NWs/P3HT hybrid film, (**d**) energy band alignment of the CdSe NWs/P3HT hybrid film under illumination. Reproduced from [60], with permission from Wiley, 2013.

Lee et al. reported the photoresponse properties of CdSe NB photoconductors [58]. The devices exhibited high response speed (<1 ms) in a wide range of light switching frequency (up to 300 Hz) and power-law dependent photocurrent on light intensity. The phenomenon of intensity-dependent response speed and photocurrent revealed the existence of various traps in the bandgap of CdSe NB. Surface passivation with SiO_2_ thin film could reduce the number of recombination centers on CdSe NB surface, resulting in an increase in the photocurrent and a decrease in the response speed. Dai et al. investigated the influence of impurity in CdSe NB on the photoresponse of photoconductors [59]. They found unintentionally doped CdSe NBs were more suitable for application in building fast and sensitive photoconductors, while intentionally doped CdSe NBs were more advantageous in constructing high gain and responsivity photoconductors. For photoconductors based on unintentionally doped CdSe NBs, the responsivity, response, and recovery time were 3060 A/W, 20 and 27 μs, respectively. While, the responsivity, response, and recovery time were 1.5 × 10^4^ A/W, 21 and 73 μs respectively, for intentionally doped CdSe NBs. The different photoresponse characteristics mainly resulted from the impurities-induced traps. Shen et al. constructed hybrid organic-inorganic photoconductors by utilizing poly(3-hexylthiophene) (P3HT) and CdSe NW as the components on both rigid and flexible substrates [60]. Figure 7a shows the schematic illustration of the hybrid device. For the hybrid photodetectors on silicon substrate at a bias of 3 V, the photocurrent was 320 nA, much higher than that of pure CdSe NWs (120 nA), while the dark current was 2.5 nA, lower than that of pure CdSe NWs (7.5 nA). Figure 7b exhibits the switching photoresponse. The response and recovery times were less than 0.1 s. The enhanced photocurrent could be attributed to: 1) the inlay of CdSe NWs into the P3HT film significantly broadened the light absorption spectrum, and 2) as shown in Figure 7c,d, the built-in field in the interface of CdSe NWs/P3HT could promote the separation of photogenerated electron-hole pairs. CdSe NWs acted as the electron-transport material, while P3HT was the hole-transport material. The decreased dark current of hybrid photodetectors arises from difficult charge transportation through the interface of CdSe NWs/P3HT under dark conditions. Flexible hybrid photodetectors fabricated on polyethylene terephthalate (PET) substrates and printing paper could successfully work under bending up to 180°, exhibiting excellent mechanical flexibility and stability.

#### 3.2.3. Phototransistors

**Figure 8:**
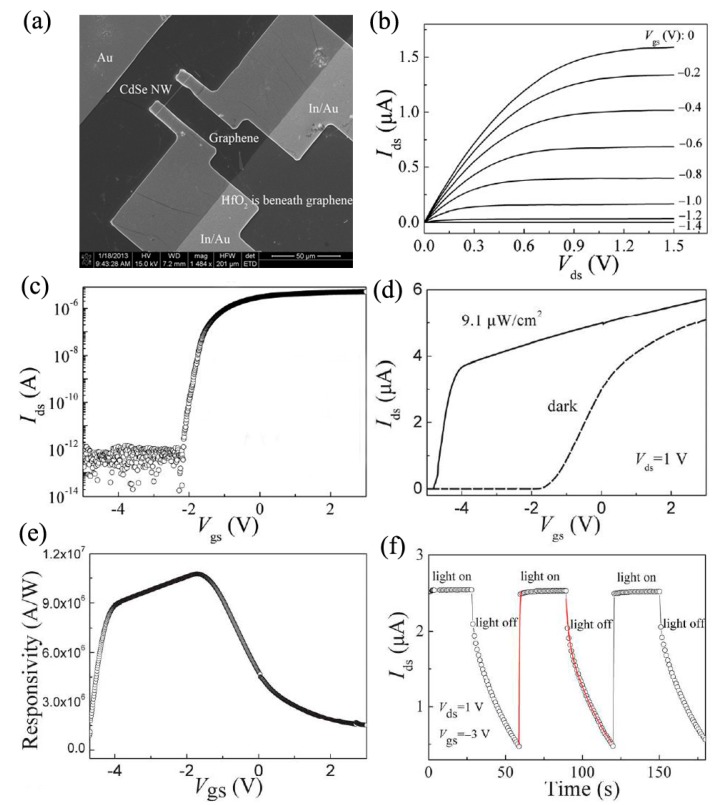
Graphene-HfO_2_-CdSe NW phototransistors: (**a**) the SEM image, (**b**) the source-drain current (*I*_ds_) versus source-drain voltage (*V*_ds_) relationships at various gate voltages (*V*_gs_), (**c**) the *I*_ds_-*V*_gs_ curve, (**d**) the *I*_ds_-*V*_gs_ curve under 633 nm light illumination (the solid line) versus in the dark (the dashed line), (**e**) the *V*_gs_ dependence of responsivity, and (**f**) the light switching response. Reproduced from [11], with permission from the Royal Society of Chemistry.

Phototransistors are a kind of three-terminal photodetectors based on the structure of either bipolar junction transistors (BJTs) or field-effect transistors (FETs) [61,62,63]. Compared to two-terminal photodetectors, the photoresponse of phototransistors can be modulated by the third terminal via either gate voltage or light intensity [64,65,66]. Dai et al. fabricated CdSe NBs-based metal-semiconductor field-effect transistors (MESFETs) and explored their application in photodetection [67]. The gate electrode was Au, which formed good Schottky contact with the CdSe NB. The threshold voltage, subthreshold swing, and on/off ratio were –0.55 V, 60.4 mV/dec, and 2 × 10^8^, respectively. Typical phototransistors showed high responsivity (~1.4 × 10^3^ A/W), high gain (~2.7 × 10^3^), and fast response speed (35–60 μs) under a gate voltage of −1 V, which was attributed to the unique advantages of the high-performance MESFET structure. Our group reported novel graphene-oxide-semiconductor (GOS) NW phototransistors, in which monolayer graphene, HfO_2_, and CdSe NW were used as top gate electrode, gate dielectric, and channel, respectively [11]. Figure 8a is the SEM image of a typical device. In dark conditions, the devices exhibited clear field effect characteristics, as seen in Figure 8b,c. The gate voltage had a remarkable modulation effect on the responsivity of such phototransistors, as shown in Figure 8e. Typical phototransistors have high responsivity (1.06 × 10^7^ A/W), gain (1.93 × 10^7^ A/W), and specific detectivity (9.68 × 10^15^ Jones) under 633 nm light illumination, which were among the highest reported values for NW-based photodetectors and exhibited a potential application in weak light detecting. The switching photoresponse of a typical phototransistor is shown in Figure 8f. The high performance of the GOS NW phototransistors originated from the following factors: 1) high transparency of graphene gate could improve light transmission, 2) a proper negative gate voltage could greatly reduce the dark current and the recombination of the photogenerated electron-hole pairs, and 3) the existence of trap states in CdSe NWs could prolong the lifetime of minority carriers and improve the gain and responsivity.

#### 3.2.4. Some New Mechanisms

In order to further improve the photoresponse characteristics or add novel functionalities of photodetectors based on 1D CdSe nanomaterials, some new mechanisms have been proposed, including enhancement effect of localized surface plasmon, optical quenching effect of photoconductivity, and piezo-phototronic effect, etc. 

**Figure 9:**
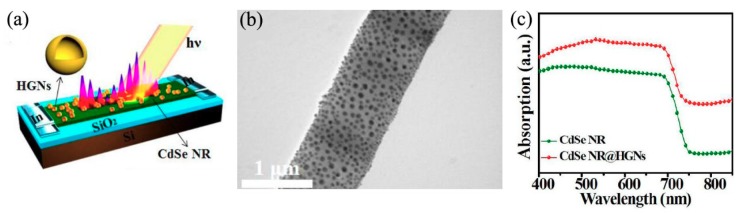
(**a**) Schematic diagram of plasmonic photodetectors, (**b**) TEM image of a CdSe NR decorated with hollow gold nanoparticles (HGNs), (**c**) absorption spectrum of both pure CdSe NR and decorated with HGNs. Reproduced from [71], with permission from Optical Society of America, 2015.

Localized surface plasmon (LSP), which is the collective oscillation of electrons in noble metal nanoparticles excited by light at an appropriate wavelength, can result in a remarkable localized-field enhancement effect and can be used to improve light absorbance in photodetectors [68,69,70,71]. Luo et al. constructed plasmonic photodetectors based on CdSe nanoribbons (NRs) decorated with hollow gold nanoparticles (HGNs) [71]. Figure 9a showed the schematic diagram, and Figure 9b was the TEM image of a CdSe NR decorated with HGNs. The resulting LSP could enhance the absorption of incident light in CdSe NBs, as seen in Figure 9c. After decoration with HGNs, the responsivity could be improved from 2.75 × 10^4^ to 4.87 × 10^4^A/W, and the detectivity increased from 3.18 × 10^13^ to 7.5 × 10^13^ Jones, respectively. Besides, the LSP resonance peaks could be rationally tuned via adjusting the geometric dimensions of metal nanoparticles, which was an advantage for designing photodetectors with spectral selectivity.

**Figure 10:**
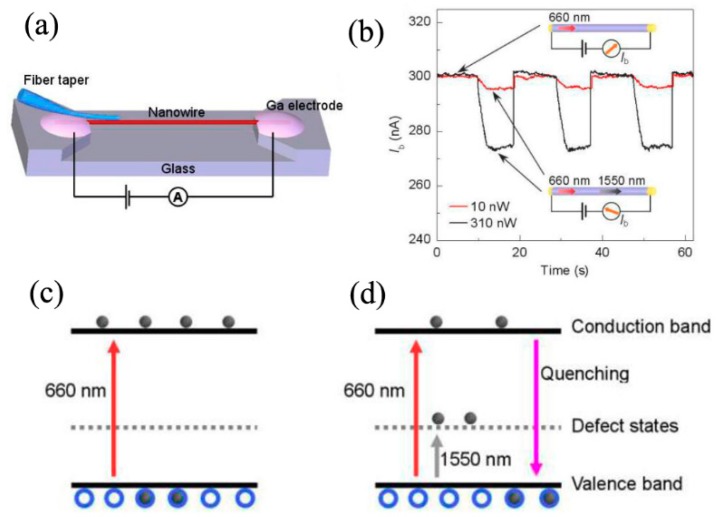
(**a**) Schematic diagram for investigating the quenching effect of photoconductivity in a single CdSe NW, (**b**) the experimental results of optical quenching effect in a CdSe NW, the energy band diagram of CdSe NWs under illumination of (**c**) above-bandgap light (660 nm) and (**d**) both above-bandgap light (660 nm) and sub-bandgap (1550 nm) light. Reproduced from [72], with permission from Optical Society of America, 2011.

Optical quenching of photoconductivity refers to the phenomenon where sub-bandgap light can reduce the background photoconductivity established by above-bandgap light illumination, which has potential application for sub-bandgap infrared photodetection [72,73,74]. Tong et al. reported the optical quenching of photoconductivity in single CdSe NW with sub-bandgap excitation power down to pW level at room temperature [72]. Figure 10a was the schematic diagram for investigating the quenching effect of photoconductivity in a single CdSe NW. The CdSe NW was suspended across a glass channel, with gallium coated at both ends of CdSe NW as electrodes. Excitation light was evanescently coupled into the CdSe NW with a fiber taper. Figure 10b was the experimental results. Based on the energy band diagram of CdSe NWs, as shown in Figure 10c,d, the mechanism of optical quenching of photoconductivity could be understood as follows: 1) the background photocurrent was generated due to the illumination of above-bandgap (660 nm) light, 2) upon the illumination of sub-bandgap (1550 nm) light, electrons would be excited from the valence band to the defect states. Then, excess holes were created and would recombine with free electrons in the conduction band, resulting in decreased conductivity. A typical responsivity of 0.5 A/W for quenching the photoconductivity was observed, under a 1550 nm laser with 10 nW power for waveguiding excitation.

**Figure 11:**
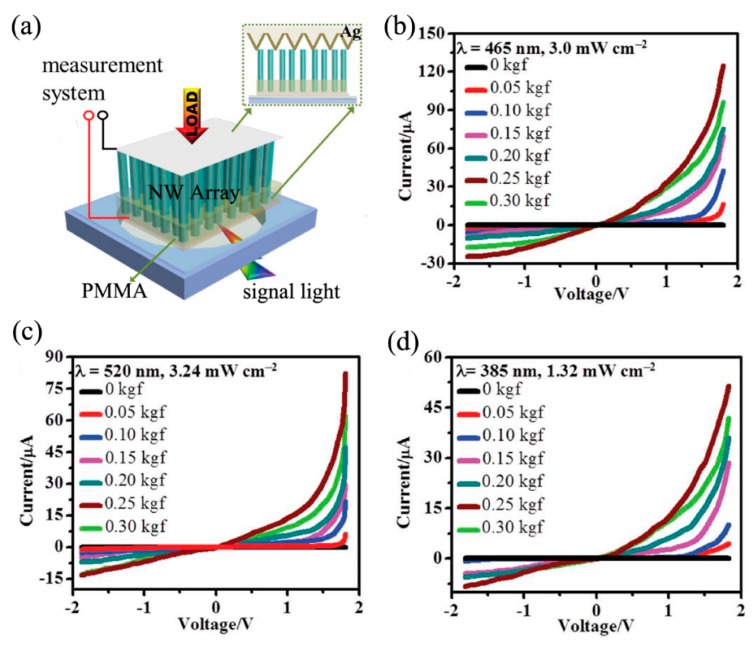
(**a**) Schematic diagram of the device configuration, and the load dependence of *I–V* curves under (**b**) blue (465 nm), (**c**) green (520 nm), and (**d**) UV (385 nm). Reproduced from [77], with permission from Wiley, 2015.

The piezo-phototronic effect refers to a coupling effect of piezoelectric polarization, optical excitation, and semiconductor properties in piezoelectric semiconductors (such as CdSe, CdS, ZnO, GaN, and MoS_2_, etc.) [75,76,77,78,79]. This effect can be utilized to enhance the photoresponse of photodetectors through creating piezoelectric polarization charges and modulating the generation, separation, transport, and/or recombination of charge carriers [80,81,82]. Zhou et al. reported a kind of broadband photodetectors based on CdSe/ZnTe core/shell NW arrays, in which the performance of photodetectors was largely boosted by the piezo-phototronic effect [77]. Figure 11a shows the schematic diagram of the photodetectors. As shown in Figure 11b–d, under UV (385 nm), blue (465 nm), and green (520 nm) light illumination, the peak photocurrent of the photodetectors is two orders higher at 0.25 kilogram force (kgf) than no load, and the resulting responsivity changed by four orders of magnitude, which was partially attributed to the piezo-phototronic effect induced by a change in the barrier height at the Ag-ZnTe Schottky junction, and in the CdSe-ZnTe type-II band alignment.

## 4. Summary and Outlook

In summary, because of unique geometrical and physical properties, 1D CdSe nanomaterials have exhibited great potential application for high-performance photodetectors. In this review, recent progresses on various synthesis methods of 1D CdSe nanomaterials and the application in photodetectors have been introduced. The bottom-up synthesis methods of 1D CdSe nanomaterials, such as VLS method, SLS method, electrochemical deposition, etc., have been analyzed in detail. Considering diverse factors, such as crystal quality, morphology, growth temperature, and cost, etc., each synthesis method has their own advantages and shortcomings. The illustration of recent advancements on photodetectors based on 1D CdSe nanomaterials has been divided into three parts based on the device configurations and working mechanisms, including photodiodes, photoconductors, and phototransistors. Besides, some new mechanisms (such as enhancement effect of localized surface plasmon, optical quenching effect of photoconductivity, and piezo-phototronic effect, etc.), which can be utilized to enhance the performance of photodetectors, have also been elaborated. The review can provide meaningful guidance for the future fabrication and design of novel nanoscale photodetectors.

Despite these progresses, there still exist some major challenges towards the practical integration and application of photodetectors based on 1D CdSe nanomaterials. First, a low-cost, simple, and reproducible method, which can be used to synthesize 1D CdSe nanomaterials with precisely controllable geometrical and physical properties, such as diameter, length, orientation, crystal quality, and doping concentration, is still lacking. Second, in most cases, controllable and scalable postgrowth transfer and assembly of 1D CdSe nanomaterials on target substrates is difficult in the fabrication of photodetectors. Up to now, some methods have been proposed, including the use of fluid-assisted alignment, the Langmuir-Blodgett technique, the dielectrophoretic method, and contact printing, etc. [83,84,85,86,87]. However, their ability to precisely control the position and orientation of each 1D CdSe nanomaterial is still inadequate. Third, due to the large surface-to-volume ratio, 1D CdSe nanomaterials have plenty of surface states, which have significant influence on the response speed, responsivity, and stability of photodetectors. Although some methods about the passivation of surface states in CdSe nanomaterials have been reported [58,88,89,90], the influence mechanism of passivation on the photoresponse characteristics of 1D CdSe nanomaterials needs to be further investigated. Finally, although 1D CdSe nanomaterials have a high absorption coefficient near the band edge, they absorb little light due to thin diameters. In order to further enhance the light absorption, some new mechanisms and structures needs to be introduced. Recently, Hu et al. summarized six routes to realize localized fields enhancement in two-dimensional materials photodetectors, including ferroelectric field, photogating electric field, floating gate-induced electric field, interlayer built-in field, localized optical field, and photo-induced temperature gradient field, respectively [91,92]. The localized fields have been proven to effectively enhance optical absorption, suppress background noise, and improve electron-hole separation efficiency. Combining 1D CdSe nanomaterials photodetectors with the idea of localized fields enhancement might be an efficient method to further improve the performance of photodetectors in the future.
